# Suppression of USP8 sensitizes cells to ferroptosis via SQSTM1/p62-mediated ferritinophagy

**DOI:** 10.1093/procel/pwac004

**Published:** 2022-10-14

**Authors:** Lihong Liu, Birong Zheng, Manhui Luo, Jie Du, Fang Yang, Chuanxin Huang, Zengyi Ma, Chunmei Li, Deyin Guo, Hong Peng

**Affiliations:** MOE Key Laboratory of Tropical Disease Control, Centre for Infection and Immunity Study (CIIS), School of Medicine, Sun Yat-sen University, Shenzhen 510275, China; MOE Key Laboratory of Tropical Disease Control, Centre for Infection and Immunity Study (CIIS), School of Medicine, Sun Yat-sen University, Shenzhen 510275, China; MOE Key Laboratory of Tropical Disease Control, Centre for Infection and Immunity Study (CIIS), School of Medicine, Sun Yat-sen University, Shenzhen 510275, China; MOE Key Laboratory of Tropical Disease Control, Centre for Infection and Immunity Study (CIIS), School of Medicine, Sun Yat-sen University, Shenzhen 510275, China; Shanghai Institute of Immunology and Department of Immunology and Microbiology, Key Laboratory of Cell Differentiation and Apoptosis of Chinese Ministry of Education, Faculty of Basic Medicine, Shanghai Jiao Tong University School of Medicine, Shanghai 200025, China; Shanghai Institute of Immunology and Department of Immunology and Microbiology, Key Laboratory of Cell Differentiation and Apoptosis of Chinese Ministry of Education, Faculty of Basic Medicine, Shanghai Jiao Tong University School of Medicine, Shanghai 200025, China; Department of Neurosurgery, Huashan Hospital, Shanghai Medical College, Fudan University, Shanghai 200433, China; MOE Key Laboratory of Tropical Disease Control, Centre for Infection and Immunity Study (CIIS), School of Medicine, Sun Yat-sen University, Shenzhen 510275, China; MOE Key Laboratory of Tropical Disease Control, Centre for Infection and Immunity Study (CIIS), School of Medicine, Sun Yat-sen University, Shenzhen 510275, China; MOE Key Laboratory of Tropical Disease Control, Centre for Infection and Immunity Study (CIIS), School of Medicine, Sun Yat-sen University, Shenzhen 510275, China

**Dear Editor**,

Ferroptosis is a newly discovered form of regulated cell death characterized by increased intracellular iron accumulation and subsequent lipid peroxidation (Dixon et al., 2012). Studies have revealed that ferroptosis plays an important role in multiple physiological and pathological processes including degenerative diseases, carcinogenesis, and cancer immunotherapy (Hassannia et al., 2019, Wang et al., 2019).

As an intracellular iron storage protein, ferritin is degraded via the selective autophagy-mediated degradation process named “ferritinophagy” to regulate iron homeostasis (Gao et al., 2016). Targeting genes related to iron homeostasis have been corroborated to modulate cellular sensitivity to ferroptosis (Alvarez et al., 2017, Protchenko et al., 2021). In consideration of an increased iron demand in cancer cells than non-cancer cells to enable growth, cancer cells are more vulnerable to iron-catalyzed ferroptosis, prompting ferroptosis induction can be employed as a promising approach in cancer therapy (Shen et al., 2014, Torti et al., 2018, Hassannia et al., 2019).

Several deubiquitinating enzymes (DUBs) including OTUB1 and BAP1 have been reported to mediate ferroptosis in human cancers (Zhang et al., 2018, Liu et al., 2019). Ubiquitin-specific peptidase 8 (USP8) is originally identified as a growth-regulated DUB that accumulates upon growth stimulation. Although USP8 depletion suppresses cell proliferation in various cancer cells and induces cell death in some cell lines (Islam et al., 2021), whether a direct connection exists between USP8 and ferroptosis remains unknown. Our previous study found that USP8 decreases autophagic flux through deubiquitinating autophagy receptor SQSTM1/p62 (sequestosome 1), thus negatively controls autophagy (Peng et al., 2019). Considering the correlation between autophagy and ferroptosis, we assume that USP8 may play a role in regulating ferroptosis.

Firstly, *USP8* knockdown (KD) mouse embryonic fibroblasts (MEFs) were constructed and confirmed by immunoblotting (Fig. 1A). Then, CCK8 cell viability assay and PI staining showed that knockdown of *USP8* sensitized cells to ferroptosis (Fig. 1B and 1C). Similar results were obtained in NCI-H1299 and HepG2 cells (Fig. S1A–C). Erastin-induced lipid peroxidation, which is the typical marker of ferroptosis, was significantly accumulated in sh*USP8* cells by flow cytometry using the fluorescent probe C11-BODIPY (Fig. 1D). Furthermore, the increased erastin-induced cell death in *USP8* KD cells can be reversed by deferoxamine (DFO, an iron-chelating agent) and ferrostatin-1 (Fer-1, a ferroptosis inhibitor) (Fig. 1E and 1F), but not apoptosis (Z-VAD-FMK) or necrosis (Nec-1s) inhibitors (Fig. 1E), suggesting that the increased erastin-induced cell death by *USP8* knockdown is a ferroptosis-dependent process. In addition, downregulation of USP8 remarkably promoted erastin-induced ferroptotic events including malondialdehyde (MDA, an end product of lipid peroxidation) production, enhanced intracellular ferrous iron level, total reactive oxygen species (ROS) and lipid ROS level (Figs. 1G–I and S1D), which could be abolished by DFO or Fer-1 treatment. These results indicated that USP8 may be a specific negative regulator of ferroptosis, instead of apoptosis or necrosis modulator.

**Figure 1.**
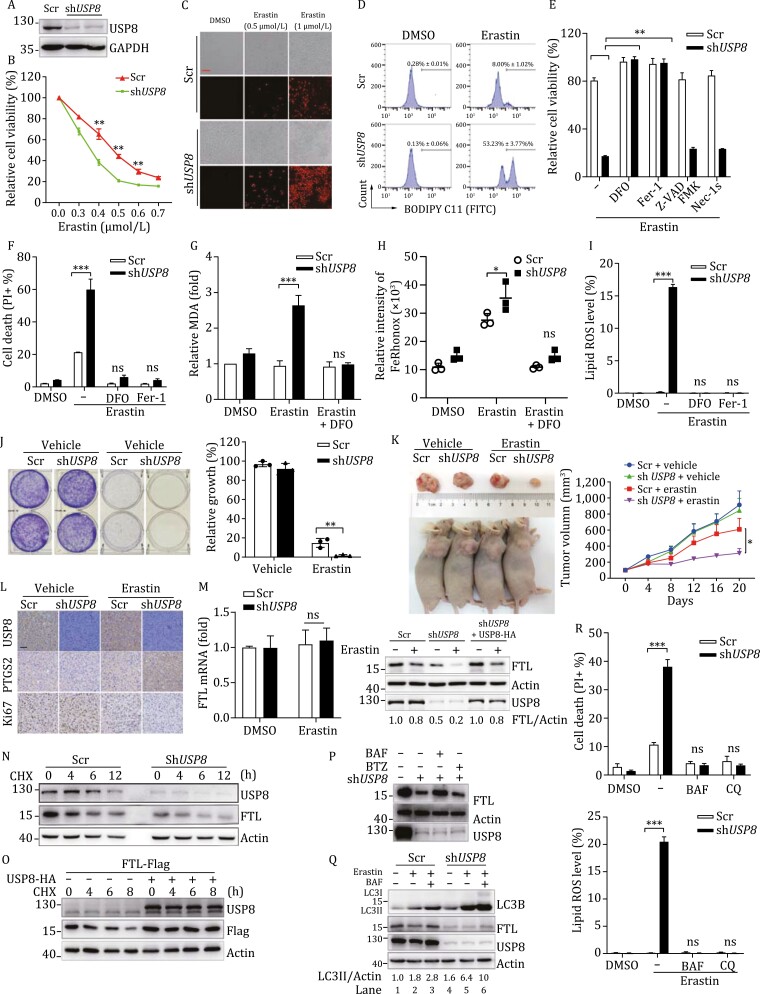
USP8 knockdown facilitates ferroptosis induced by erastin via ferritinophagy. (A) Knockdown of *USP8* in MEF cells was verified by immunoblotting. (B) MEF cells were treated with indicated erastin for 24 h, cell viability was assayed using a CCK8 kit (*n* = 3). (C) MEF cells were treated with 0.5 μmol/L or 1 μmol/L erastin for 12 h, cell death was observed using PI staining by microscopy (black and white: phase contract; red: PI staining; scale bar = 100 μm). (D) MEF cells were treated with 1 μmol/L erastin for 8h, and flow cytometric detection of lipid ROS was performed using BODIPY C11. Representative data are shown and the statistical analysis is from three independent experiments. (E) MEF cells were treated with 0.5 μmol/L erastin with or without a cell death inhibitor (DFO, 10 μmol/L; Fer-1, 1 μmol/L; ZVAD-FMK, 10 μmol/L; nec-1s, 10 μmol/L) for 24 h. Cell viability was assayed using a CCK8 kit. (F–I) Cells were treated with 1μmol/L erastin alone or with a ferroptosis inhibitor (DFO, 10 μmol/L; Fer-1, 2 μmol/L), cell death was quantified by PI staining coupled with flow cytometry (F); the lipid formation was measured by MDA assay (G); the intracellular ferrous iron level and lipid ROS level (H, I) was assessed by flow cytometry. (J) Cells were treated with erastin (5 μmol/L) for 24 h and then re-plated in the same dilutions in plates for colony formation assay. After 7 days, cells were fixed, stained and photographed. (K) The 4-week-old immunodeficient nude mice (5 mice per group) were injected subcutaneously with NCI-H1299 cells (5 × 10^6^ cells per mouse) and treated with erastin (15 mg per kg intraperitoneal, twice every other day) when the tumor volume reached for 100 mm^3^, for 20 days. Tumor volume was calculated every 4 days. (L) Staining of USP8, FTL and PTGS2 in isolated tumor at day 20 was assayed by immunohistochemistry analysis (scale bar = 100 μm). (M) HepG2 cells were treated with erastin (10 μmol/L) for 12 h, and FTL mRNA levels are assayed by qRT-PCR. Indicated cells were transfected with USP8-HA for 24 h followed by treatment with erastin for 12 h, and FTL protein level was assayed by immunoblotting. (N) HepG2 cells were treated with 10 μmol/L erastin and 50 μg/mL CHX for indicated period, protein levels were detected by immunoblotting. (O) HEK 293T cells were transfected with the indicated plasmids for 24 h and then treated with 50 μg/mL CHX for indicated period, protein levels were detected by immunoblotting. (P) HepG2 cells were treated with BAF (200 nmol/L) or BTZ (1 μmol/L) for 8 h, cell extracts were analyzed by immunoblotting using indicated antibodies. (Q) HepG2 cells were treated with erastin (10 μmol/L) for 12 h, BAF was added 8 h before cell harvest, and cell extracts were analyzed by immunoblotting using indicated antibodies. The accumulation of LC3II (faster migrating form) is indicative of the induction of autophagy. (R) Indicated MEF cells were treated with 1 μmol/L erastin alone or with autophagy inhibitor (BAF, 20 nmol/L; CQ, 50 μmol/L) for 12 h, cell death was quantified by PI staining; lipid ROS level was assessed by BODIPY C11 coupled with flow cytometry. *P ≤ 0.05, **P ≤ 0.01, ***P ≤ 0.001, ns, not significant.

To confirm whether *USP8* suppression enhances the anti-cancer activity of erastin *in vivo*, we stably knocked down *USP8* in NCI-H1299 cell line by lentiviral vector. Knockdown of USP8 significantly enhanced erastin-induced ferroptotic cell death in colony formation assay (Fig. 1J). Compared with the control shRNA group, *USP8* knockdown effectively reduced the size of tumors formed in a xenografted mouse model (Fig. 1K). Immunohistochemistry (IHC) analysis of the expression of prostaglandin-endoperoxide synthase-2 (PTGS2), a marker for the assessment of oxidative stress and ferroptosis *in vivo*, indicated the combination effect of knocking down *USP8* and erastin treatment in triggering tumor ferroptosis *in vivo* (Fig. 1L).

Ferritin is a pivotal intracellular protein that stores iron, composed of ferritin light chain (FTL) and ferritin heavy chain 1 (FTH1), which protects the cell from free iron participating in the generation of free radicals via Fenton-like reactions (Dixon et al., 2014). Considering USP8 is a deubiquitination enzyme, we tested whether FTL, a substrate of ferritinophagy, is degraded in USP8-regulated ferroptosis. Knockdown of *USP8* did not influence the expression of FTL mRNA, whereas it remarkably promoted ferritin degradation in erastin-induced HepG2 cells, and this effect was rescued by RNAi-resistant USP8 expression (Fig. 1M). Consistently, in the presence of cycloheximide (CHX, an inhibitor of protein translation), inhibition of *USP8* promoted the degradation of endogenous FTL protein in human liver cancer cell line HepG2 (Figs. 1N and S2A) and overexpression of USP8 delayed the protein turnover of exogenous FTL protein in HEK 293T cells (Figs. 1O and S2B). The decrease of ferritin in sh*USP8* cells was partly abrogated by bafilomycin A1 (BAF, an autophagy inhibitor) but not bortezomib (BTZ, a proteasome inhibitor) (Fig. 1P), indicating that USP8 knockdown destabilized ferritin through autophagy-lysosome degradation pathway. Ferritin is delivered to lysosome for degradation via autophagy, which was referred to as “ferritinophagy” (Mancias et al., 2014). We assumed that the regulation of ferritin mediated by *USP8* is an autophagy-related process. Then, we treated the control and sh*USP8* cells with erastin alone or in the presence of BAF and detected the state of LC3, which is a typical marker of autophagy process. Erastin treatment significantly promoted the conversion from LC3I to LC3II in sh*USP8* cells, this phenomenon was even more remarkable in the presence of BAF (Fig. 1Q). In addition, the increased autophagic flux event was also observed in sh*USP8* cells (Fig. S3). To further confirm the role of ferritinophagy in USP8-mediated ferroptosis, we exploited two typical pharmacological inhibitors of autophagy: BAF and chloroquine (CQ) to block the autophagy. As expected, both BAF and CQ treatments significantly attenuated erastin-induced cell death and lipid ROS formation in sh*USP8* MEFs (Fig. 1R). Taken together, these data indicate that downregulation of USP8 triggered ferritinophagy during ferroptosis and promoted the degradation of ferritin.

Recently, USP8 was proved to be a negative regulator of autophagy by deubiquitinating SQSTM1 (Peng et al., 2019). To explore the function of SQSTM1 in USP8-induced ferritinophagy, we generated *USP8*/*SQSTM1* double knockdown HepG2 cell lines (Fig. 2A). The results showed that erastin-induced cell death in sh*USP8* cells was reversed by concurrent knockdown of *SQSTM1* (Figs. 2B, 2C and S4). Moreover, we constructed *SQSTM1* knockdown HepG2 cells as control, sh*SQSTM1* cells were resistant to elastin-induced ferroptosis, and knockdown of *ATG7* or *SQSTM1* greatly reduced the sensitivity of the sh*USP8* MEF cells to erastin-induced ferroptosis and decreased lipid ROS level (Fig. 2C and 2D), while reconstituting SQSTM1 in sh*USP8*/*shSQSTM1* MEF cells partially restored cellular ferroptosis sensitivity (Fig. 2D), indicating USP8-mediated ferroptosis is an autophagy-dependent process involving SQSTM1 participation.

**Figure 2.**
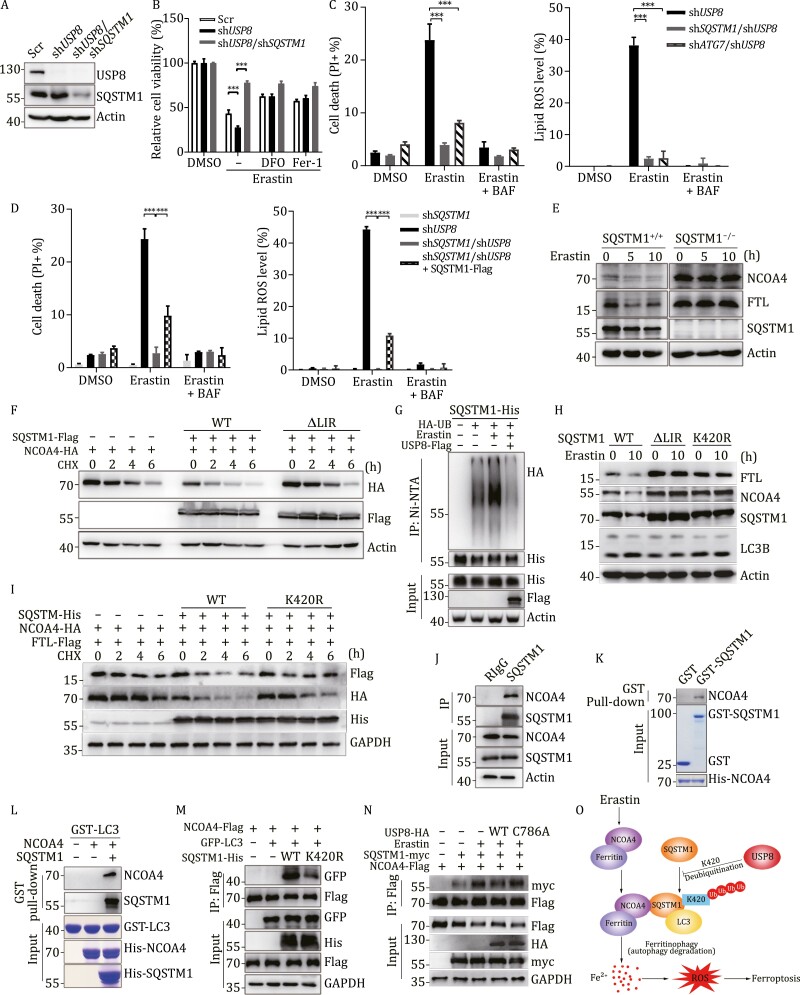
The interaction between LC3 and NCOA4 mediated by SQSTM1 is involved in USP8-mediated ferritinophagy. (A) Knockdown of *USP8* and *SQSTM1* in HepG2 cells by indicated shRNA was confirmed with immunoblotting. (B) Indicated HepG2 cells were treated with erastin (10 μmol/L) alone or in the presence with DFO or Fer-1, cell viability was quantified with a CCK8 kit. (C) Indicated MEF cells were treated with 1 μmol/L erastin alone or with BAF (20 nmol/L) for 12 h, cell death and lipid ROS were assessed by flow cytometry. (D) Indicated MEF cells were transfected with empty vector or SQSTM1-Flag for 24 h, and then treated with 1 μmol/L erastin alone or with BAF (20 nmol/L) for 12 h, cell death and lipid ROS level was assessed by flow cytometry. (E) Wild-type MEF and *SQSTM1*^−/−^ MEF cells were treated with 1 μmol/L erastin for 5 and 10 h, indicated protein level was assayed by immunoblotting. (F) HEK 293T cells were transfected with NCOA4-HA and SQSTM1-Flag wild type or SQSTM1_∆LIR_-Flag for 24 h, then cells were treated CHX (50 μg/mL) for indicated time before cell harvest, and protein levels were determined by immunoblotting. (G) HEK293T cells were co-transfected with SQSTM1-His, HA-UB and USP8-flag for 24 h, followed by treatment with 10 μmol/L erastin for 12 h. The cell lysates were subjected to pull down using the Ni^2+^-NTA beads under denaturation conditions, followed by immunoblotting. (H) *SQSTM1*^−/−^ MEF cells were transfected with wild type SQSTM1, or SQSTM1_K420R_ mutant plasmids via electroporation, 24 h after transfection, cells were treated with 10 μmol/L erastin for 10 h, indicated protein level was assayed by immunoblotting. (I) HEK293T cells were transfected with NCOA4-HA and FTL-Flag and SQSTM1-His wild type or SQSTM1_K420R_-Flag for 24 h, then cells were treated CHX (50 μg/mL) for indicated time before cell harvest, and protein levels were determined by immunoblotting. (J) HepG2 cells were harvested and immunoprecipitated with anti-SQSTM1 antibody, followed by immunoblotting using indicated antibodies. (K) Purified fusion proteins GST-SQSTM1 and His-NCOA4 were used for the *in vitro* GST pull down assay, GST protein was used as negative control. His-NCOA4 was incubated with GST or GST-SQSTM1 overnight, and then the protein was detected by immunoblotting. (L) Purified His-NCOA4 was incubated with purified GST-LC3 in the absence or presence with His-SQSTM1 in GST pulldown assay and then the protein was detected by immunoblotting. (M) HEK293T cells were co-transfected with GFP-LC3, NCOA4-flag and SQSTM1-His wild type or K420R mutant for 36 h, the cell lysates were immunoprecipitated with flag beads and immunoblotted with antibodies against GFP, His and Flag. (N) HEK293T cells were co-transfected with SQSTM1-myc, NCOA4-flag and USP8-HA wild type or C786A mutant for 24 h and then treated with 10 μmol/L erastin for 12 h, the cell lysates were immunoprecipitated with flag beads and immunoblotted with indicated antibodies. (O) Schematic diagram depicting the regulation of USP8 in ferroptosis. Inhibition of USP8-SQSTM1-NCOA4-ferritin axis facilities the sensitivity of cancer cell to ferroptosis through regulating cellular iron level, which is triggered through the initiation of ferritinophagy derived from SQSTM1-mediated autophagy. *P ≤ 0.05, **P ≤ 0.01, ***P ≤ 0.001, ns, not significant.

As two important marker proteins of ferritinophagy, nuclear receptor coactivator 4 (NCOA4) and FTL are always degraded under ferroptosis, which may be regulated by SQSTM1. In accordance to our expectation, depletion of SQSTM1 almost abolished erastin-mediated downregulation of endogenous NCOA4 and FTL (Fig. 2E). In addition, overexpression of wild type SQSTM1 dramatically promoted the degradation of NCOA4, but not the LIR (LC3-interacting region) deletion mutant SQSTM1ΔLIR, which is deficient in mediating selective autophagy (Figs. 2F and S6A). Previous studies have proved that ubiquitination of SQSTM1 promotes its activity as autophagic receptor and USP8 removes the ubiquitination of SQSTM1 mainly at K420 position to suppress autophagic flux (Peng et al., 2019). Then we observed increased ubiquitination of SQSTM1 under erastin treatment, which was significantly reduced by USP8, suggesting USP8-regulated SQSTM1 ubiquitination was involved in USP8-mediated ferroptosis (Fig. 2G). Interestingly, we also detected increased ubiquitination of SQSTM1 under iron overload induced by ferric ammonium citrate (FAC) (Fig. S5), implying SQSTM1 may be an iron sensor protein in response to iron overload stress. In order to further prove SQSTM1 is vital to ferritinophagy, we re-transfected wild type SQSTM1, SQSTM1ΔLIR, or SQSTM1_K420R_ into *SQSTM1*^−/−^ MEF cells, and then treated with erastin. The results showed that protein level of NCOA4 and FTL only decreased in wild-type SQSTM1-expressing cells, but not in SQSTM1ΔLIR and SQSTM1_K420R_ cells, and the protein level of endogenous LC3II only increased in wild-type SQSTM1 reconstituted cells (Fig. 2H). Furthermore, compared with wild-type SQSTM1, the introduction of SQSTM1_K420R_ attenuated the degradation of NCOA4 and FTL, suggesting that ubiquitination of SQSTM1 is critical to promoting the degradation of these two proteins (Figs. 2I and S6B).

Accordingly, we propose that USP8-regulated ferroptosis is SQSTM1-dependent and SQSTM1’s function as autophagic receptor plays a crucial role in aiding this process. To verify this hypothesis, we executed endogenous Co-IP assay in HepG2 cells and verified the interaction between SQSTM1 and NCOA4 (Fig. 2J). Results of exogeneous Co-IP assay in HEK 293T cells indicated that neither PB1 or UBA domain of SQSTM1 participated in the interaction with NCOA4 implying that the middle fragment of SQSTM1(103–338 aa) mediates the interaction with NCOA4 (Fig. S7A). Besides that, both N (1–238 aa) and C (239–614 aa) fragments of NCOA4 interact with SQSTM1 (Fig. S7B). Results of fluorescent image also represented the colocalization between SQSTM1 and NCOA4 (Fig. S8A). In *in vitro* GST pull-down assay, his-NCOA4 was detected only in the GST-SQSTM1 affinity column, suggesting a direct interaction between the two proteins (Fig. 2K). Although NCOA4 is regarded as a ferritinophagy receptor, there is no direct evidence of interaction between NCOA4 and LC3. As NCOA4 does not have a canonical LC3-interacting region motif (Mancias et al., 2014), we suspected that SQSTM1 might bridge the binding of NCOA4 with LC3 during ferritinophagy. To test this hypothesis, we evaluated whether NCOA4 could be pulled down with LC3 by incubating with or without SQSTM1. As showed in Fig. 2L, NCOA4 specifically interacted with LC3 only when SQSTM1 was added, proving that SQSTM1 was necessary for NCOA4-LC3 recognition. Meanwhile, results of immunostaining showed that NCOA4 colocalized with LC3 much stronger in the presence of SQSTM1 (Fig. S8B). In addition, the NCOA4-LC3 interaction mediated by SQSTM1 was confirmed *in vivo,* this interaction significantly decreased when SQSTM1_K420R_ mutant was co-transfected (Fig. 2M), implying the ubiquitylation of K420 is vital to the interaction between NCOA4 and LC3 *in vivo*. Ubiquitination is the typical modification of autophagic substrates, and enhanced ubiquitination modification of NCOA4 was observed under erastin treatment (Fig. S9). Furthermore, the interaction between NCOA4 and SQSTM1 was dramatically enhanced under erastin treatment, which could be negatively modulated by wild type USP8 but not deubiquitinase activity-deficient mutant C786A, indicating the deubiquitinase activity of USP8 is essential to ferroptosis regulation (Fig. 2N). Taking all these data into consideration, we conclude that SQSTM1 acts as a platform, tethering NCOA4 to LC3 during the process of ferritinophagy.

In summary, we report that USP8 inhibition promotes ferroptosis by promoting ferritin degradation in cancer cells. Mechanistically, autophagy receptor SQSTM1/p62 acts as a platform to promote the interaction between NCOA4 and LC3, thus promoting ferritin degradation to enhance ferroptosis sensitivity, this process is regulated by USP8 (Fig. 2O). The suppression of USP8-SQSTM1-NCOA4-ferritin axis through up-regulating ferritinophagy and intracellular iron levels sensitized cancer cells to ferroptosis. These results indicate that USP8 plays a major role in modulating ferritinophagy and ferroptotic responses in cancer cells and reveal that USP8 may be a potential target in ferroptosis-mediated cancer therapy. Moreover, two research groups have reported *USP8* mutations in pituitary tumors results in adrenocorticotropic hormone (ATCH) over-secretion (Ma et al., 2015). Our current data imply that serum ferritin may be used as a new biomarker for early diagnosis for Cushing’s disease patients with *USP8* mutation, which need further explorations.

## Supplementary Material

pwac004_suppl_Supplementary_Material

## Footnotes

This work was supported by the National Natural Science Foundation of China Grant Nos. 81620108020, 32041002, to D.G.; Grant Nos. 81702724 to H.Peng); Guangdong Zhujiang Talents Program to (D.G.); Shenzhen Science and Technology Program (JSGG20200225150431472 and KQTD20180411143323605 to D.G.; JCYJ20190807161009621 to H.Peng) and National Ten-thousand Talents Program (to D.G.). No potential conflict of interest was reported by the authors. Animal experiments were approved by the Institutional Animal Care and Use Committee of Sun Yat-Sen University. LL, BZ and ML performed the experiments and analyzed the data. JD, FY and ZM participated in the data and the mice xenograft work. CH and CL helped with the manuscript writing. HP, DG and LL designed the experiments and wrote the manuscript. All authors read and approved the final paper.

## References

[CIT0001] Alvarez SW , Sviderskiy VO, Terzi EM et al. NFS1 undergoes positive selection in lung tumours and protects cells from ferroptosis. Nature 2017;551(7682):639–43. 29168506 10.1038/nature24637 PMC5808442

[CIT0002] Dixon SJ , Lemberg KM, Lamprecht MR et al. Ferroptosis: an iron-dependent form of nonapoptotic cell death. Cell 2012;149(5):1060–72. 22632970 10.1016/j.cell.2012.03.042 PMC3367386

[CIT0003] Dixon SJ , Stockwell BR. The role of iron and reactive oxygen species in cell death. Nat Chem Biol 2014;10(1):9–17. 24346035 10.1038/nchembio.1416

[CIT0004] Gao M , Monian P, Pan Q et al. Ferroptosis is an autophagic cell death process. Cell Res 2016;26(9):1021–32. 27514700 10.1038/cr.2016.95 PMC5034113

[CIT0005] Hassannia B , Vandenabeele P, Vanden Berghe T. Targeting ferroptosis to iron out cancer. Cancer Cell 2019;35(6):830–49. 31105042 10.1016/j.ccell.2019.04.002

[CIT0006] Islam MT , Chen F, Chen H. The oncogenic role of ubiquitin specific peptidase (USP8) and its signaling pathways targeting for cancer therapeutics. Arch Biochem Biophys 2021;701:108811. 33600786 10.1016/j.abb.2021.108811

[CIT0007] Liu T , Jiang L, Tavana O, Gu W. The deubiquitylase OTUB1 mediates ferroptosis via stabilization of SLC7A11. Cancer Res 2019;79(8):1913–24. 30709928 10.1158/0008-5472.CAN-18-3037 PMC6467774

[CIT0008] Ma ZY , Song ZJ, Chen JH et al. Recurrent gain-of-function USP8 mutations in Cushing’s disease. Cell Res 2015;25(3):306–17. 25675982 10.1038/cr.2015.20 PMC4349249

[CIT0009] Mancias JD , Wang X, Gygi SP et al. Quantitative proteomics identifies NCOA4 as the cargo receptor mediating ferritinophagy. Nature 2014;509(7498):105–109. 24695223 10.1038/nature13148 PMC4180099

[CIT0010] Peng H , Yang F, Hu Q et al. The ubiquitin-specific protease USP8 directly deubiquitinates SQSTM1/p62 to suppress its autophagic activity. Autophagy 2019;1–11. 10.1080/15548627.2019.1635381 PMC7138243 31241013

[CIT0011] Protchenko O , Baratz E, Jadhav S et al. Iron chaperone poly rC binding protein 1 protects mouse liver from lipid peroxidation and steatosis. Hepatology (Baltimore, Md.) 2021;17(3):1176–1193. 10.1002/hep.31328 PMC8364740 32438524

[CIT0012] Shen J , Sheng X, Chang Z et al. Iron metabolism regulates p53 signaling through direct heme-p53 interaction and modulation of p53 localization, stability, and function. Cell Rep 2014;7(1):180–193. 24685134 10.1016/j.celrep.2014.02.042 PMC4219651

[CIT0013] Torti SV , DHM, Paul BT, Blanchette-Farra N, Torti FM. Iron and cancer. Annu Rev Nutr 2018;38:97–125. 30130469 10.1146/annurev-nutr-082117-051732 PMC8118195

[CIT0014] Wang W , Green M, Choi JE et al. CD8(+) T cells regulate tumour ferroptosis during cancer immunotherapy. Nature 2019;569(7755):270–74. 31043744 10.1038/s41586-019-1170-y PMC6533917

[CIT0015] Zhang Y , Shi J, Liu X et al. BAP1 links metabolic regulation of ferroptosis to tumour suppression. Nat Cell Biol 2018;20(10):1181–1192. 30202049 10.1038/s41556-018-0178-0 PMC6170713

