# Supplementary material for: Suppression of USP8 sensitizes cells to ferroptosis via SQSTM1/p62-mediated ferritinophagy
Source: Protein Cell. 2022 Oct 14;14(3):230–4. doi: 10.1093/procel/pwac004 (PMC10098033; doi:10.1093/procel/pwac004)
Supplement: pwac004_suppl_Supplementary_Material [file pwac004_suppl_supplementary_material.pdf]

SUPPLEMENTARY MATERIALS AND METHODS

Cell culture and transfection

HEK293T, MEF and HepG2 cells were cultured in Dulbecco's modified Eagle's medium (DMEM; 11965118, Thermo Fisher, TX, USA) supplemented with 10% fetal bovine serum and 1% penicillin-streptomycin. NCI-H1299 were cultured in RPMI medium (61870127, Thermo Fisher) supplemented with 10% fetal bovine serum and 1% penicillin-streptomycin. All cell lines were cultured in a humidified atmosphere containing 5% CO_2 at 37 ℃. Transient transfection of HEK293T cells were conducted with PEI (24765, Polyscience, Inc., IL, USA) according to the manufacturer's recommendations. Transient transfection of MEF cells and HepG2 cells were performed by using electroporation via Amaxa® Nucleofector® Technology of Lonza company, according to the manufacturer's instructions.

Antibodies and reagents

The primary antibodies used in the study were as follows: USP8 (SAB4200527, Sigma-Aldrich, MO, USA), FTL (10727-1-AP, Proteintech, Wuhan, China), Actin (66009-1-Ig, Proteintech), Flag (F1804, Sigma-Aldrich), LC3B (L7543, Sigma-Aldrich), NCOA4 (SAB1404569, Sigma-Aldrich), SQSTM1 (ab91526, Abcam, Cambridge UK), HA (SAB1411738, Sigma-Aldrich), His (SAB2702218, Sigma-Aldrich), GFP (66002-1-Ig, Proteintech), GAPDH (60004-1-Ig, Proteintech), GST (10000-0-AP, Proteintech). HRP-labelled secondary antibodies were purchased from Thermo Fisher. Erastin (S7242), Ferrostatin-1 (Fer-1, S7243), Deferoxamine

mesylate (DFO, S5742), bortezomib (PS-341, S1013), Nec-1s (S8641) and Z-VAD-FMK (S7023) were obtained from Selleck (TX, USA). Bafilomycin A1 (BAF, 196000) and Cycloheximide (CHX, C0934) were obtained from Sigma-Aldrich.

Plasmids

The human USP8 cDNA was subcloned in pcDNA3.0 with a Flag or HA tag. The human NCOA4 cDNA were subcloned in pcDNA3.0 with a HA or Flag tag, pET28a with His tag or pGEX-4T-1 vector. The human SQSTM1 cDNA was subcloned in pcDNA3.0 with a His or HA tag, point mutation and truncations of SQSTM1 were created using the Quick Change site-directed mutagenesis method with KOD Plus (Code No. KOD-201, Osaka, Japan). The GFP-tagged or GST-tagged LC3 were constructed by cloning cDNA in pcDNA3.0 or pGEX-4T-1 vector. HA- wild-type Ub were purchased from Addgene. All constructs were confirmed by DNA sequencing.

Total ROS assay.

Cellular ROS level was analyzed by flow cytometry using Reactive Oxygen Species Assay (ROS) Kit (50101ES01, Yeasen, Shanghai, China) according to the manufacturer's instructions. Cells were seeded at a density of 1×10^5 cells per well in 12-well plates and cultured overnight. Cells were exposed to indicated reagents for 12 h, then replaced with FBS-free medium containing 10 μM DCFH-DA and cultured for 45min at 37 ℃ in the dark. Cells were harvested and washed twice with DPBS to remove DCFH-DA and resuspended in 300 μl medium. The ROS level was evaluated using the flow cytometry with 488 nm excitation. A minimum of 10 000 single cells

were analyzed per well.

Cell viability assay.

Cell viability was evaluated using the cell counting kit-8 (CCK-8) (B34302, Bimake, TX, USA) according to the manufacturer's instructions. Briefly, cells were seeded in 96-well plates at a density of 1×10^4 cells per well, and treated with various concentrations of the compounds for indicated times. Then, the CCK-8 reagent (10 μl) was added to each well and incubated for 2 h at a 37 ℃, 5% CO_2 incubator. The absorbance at 450 nm was measured using a microplate reader. All experiments were carried out in triplicate.

Lipid ROS assay.

Lipid ROS level was analyzed by flow cytometry using BODIPY-C11 Dye (D3861, Thermo Fisher). Cells were seeded at a density of 1×10^5 cells per well in 12-well plates and cultured overnight. Cells were treated with indicated reagents for 8 h and then the culture medium was replaced with 1 ml medium containing 5 μM of BODIPY-C11. The plates were incubated for 20 min at 37 ℃ without light. Cells were harvested and washed twice with DPBS, then resuspended in 300 μl medium followed by flow cytometric analysis.

PI staining assay

Cell death was assessed by propidium iodide (PI, P4170, Sigma-Aldrich) staining using fluorescence microscopy and flow cytometry. Cells were seeded at a density of

2.5×10^5 cells per well in 12-well plates and cultured overnight. Cells were exposed to indicated reagents for 12 h or 24 h. For microscopy scanning, plates were washed twice with DPBS and stained with FBS-free medium containing 0.5 μM PI for 5-10 min at RT. Then dead cells were observed using fluorescence microscopy. For flow cytometry analysis, cells were collected and washed twice with DPBS, followed by staining with 0.5 μM PI for 5-10 min at RT. Cell suspension was immediately subjected to the flow cytometry to analyze dead cells. A minimum of 10 000 single cells were analyzed per well.

Malondialdehyde (MDA) assay

The relative MDA concentration in cell lysates was assessed using a Lipid Peroxidation (MDA) assay kit (#ab118970, Abcam) according to the manufacturer's instructions. Briefly, cells were collected and lysed, then cell lysates were reacted with thiobarbituric acid (TBA) at 95 ℃ for 60 minutes to generate MDA-TBA adduct. The TBA-MDA adducts were quantified colorimetrically by measuring the optical density at 532 nm.

Intracellular iron assay

Intracellular ferrous ions (Fe2+) were measured using BioTracker™ 575 Red Fe2+ Dye

(SCT030, Merck Millipore). Briefly, Cells were seeded at a density of 1×105 cells per well in 12-well plates and cultured overnight. Cells were treated with indicated reagents for 24h. Collected cells were washed with HBSS buffer and stained with 5

μM BioTracker™ 575 Red Fe^{2+} Dye in HBSS for 1 hour in a CO_2 incubator. Then cells were washed with HBSS and dissolved in 300 μl of HBSS followed by flow cytometry analysis.

RNA interference

Cells were infected with lentiviruses expressing shRNAs. Lentivirus vectors encoding shRNA against *USP8* were generated using the pLKO.1 vector with puromycin selection marker (Addgene, 8453; deposited by Dr. Bob Weinberg). shRNA against *SQSTM1* and *ATG7* were generated with pLKO.1 vector with hygromycin selection marker. For lentivirus production, HEK293T cells were seeded in 60 mm dishes. When cells reached 70-80% confluent, cells were transfected indicated plasmids using the PEI and replaced with fresh medium 6 hours post transfection. Viral particles were collected 48 h after transfection, filtered with a 0.45 μm sterile filter. 1 ml of virus supernatant was used to infect indicated cells in a 60 mm dish. Three days post-infection, cells was selected in the presence of puromycin (1 μg/ml) or hygromycin (500 μg/ml) for 5 days. Immunoblotting were performed to analyze the efficiency of gene knockdown.

Immunoprecipitation and Immunoblotting

For immunoprecipitation (IP), cells were collected 36 h post transfection and lysed in IP buffer containing 50 mM Tris-HCl, pH 7.4, 150 mM NaCl,50 mM EDTA, 10 mM NaF, 10% glycerol, 1% Triton X-100 and protease inhibitor cocktail (P8340, Sigma-Aldrich) for 30 min on ice. After centrifugation for 10 min at 15000 g, 4 ℃,

supernatants were collected and incubated with anti-FLAG® M2 Agarose Affinity Gel (A2220, Sigma-Aldrich) at 4 ℃. After 8 h of incubation, beads were washed 3 times with IP buffer. Immunoprecipitants were eluted by boiling with 2× SDS-PAGE loading buffer and prepared for immunoblotting. For immunoblotting, immunoprecipitated proteins or whole cell lysates were resolved by SDS-PAGE loading buffer. The proteins in the gel were transferred to PVDF membranes (ISEQ00010, Millipore, MA, USA). The membranes were blocked with 5% non-fat milk and then probed with indicated primary antibodies following by HRP-conjugated secondary antibodies. After three washes, the membranes were subjected to chemiluminescence using Clarity™ Western ECL Substrate (#1705060, BioRad, CA, USA). The intensities of protein bands were quantitated using ImageJ.

RNA extraction and real-time quantitative PCR (qRT-PCR)

Total RNA was extracted using the TRIzol reagent (15596018, Invitrogen,) according to the manufacturer's instructions. RNA was converted into cDNA using PrimeScript™ RT Master Mix (RR036A, Takara, Dalian, China). qRT-PCR was performed by using PowerUp™ SYBR® Green Master Mix (A25742, ABI, MA, USA) with a Applied Biosystems QuantStudio 5 Real-Time PCR Systems. The reactions were run in triplicate. The gene expression levels were normalized to actin. Data were analyzed using the relative quantification ($2^{-\Delta\Delta CT}$) method. The primers sequences used for qRT-PCR were listed in Supplementary Table S1.

Protein purification and GST pull down assay

The expression plasmids were transformed into *E.coli* BL21(DE3) cells. Protein expression was induced with 0.1 mM isopropyl β-D-1-thiogalactopyranoside (IPTG, I6758, Sigma-Aldrich) for 16 hours at 16 ℃. Bacterial pellets were harvested by centrifugation, resuspended and disrupted by sonication. GST-fusion protein was purified by using glutathione Sepharose 4B beads (17075601, GE Healthcare, IL, USA), His-tag protein was purified by Ni-NTA Agarose (30210, Qiagen, Hilden, Germany). Purified proteins were frozen at -80 ℃ for GST-pulldowns as below.

Recombinant GST fusion proteins were incubated with 20 μL of a 50%(vol/vol) slurry of glutathione Sepharose 4B beads for 30 min in a rotator at 4 ℃. And then mixed with purified proteins or cell lysate as indicated in experiments. The assay mix was incubated overnight at 4 ℃, and beads were washed three times with 1 ml wash buffer. Then proteins were eluted with 2 × SDS loading buffer and analyzed by 8-15% gradient SDS-PAGE followed by Coomassie Blue stain or immunoblotting.

Cyto-ID® autophagy detection assay

CYTO-ID® Autophagy Detection kit (ENZ-51031) was obtained from Enzo Life Sciences, Inc. and the assay was conducted according to the manufacturer's protocol. Briefly, cells were treated with indicated reagents and collected. Then cells were washed and stained with CYTO-ID for 30 min at 37 ℃ in the dark. After treatment, cells were washed and re-suspended to analyze by a flow cytometer.

***In vivo* ubiquitination assay**

HEK293T cells were transfected with indicated plasmids and harvested at 36 h after

transfection. 1/5 cells were used for direct immunoblotting and the rest of cells were collected for denaturing immunoprecipitations. Cells were lysed in denaturing buffer (6 M Guanidine HCl, 0.1 M Na_2HPO_4/NaH_2PO_4 buffer, 0.01 M Tris-HCl (pH8.0), 5 mM imidazole, 10 mM 2-mercaptoethanol (β-ME) and incubated overnight with Ni-NTA Agarose (30210, Qiagen). Then, beads were washed with buffer B (8 M urea, 0.1 M Na_2HPO_4/NaH_2PO_4, 0.01 M Tris-HCl (pH8.0), 10 mM β-ME); buffer C (8 M urea, 0.1 M Na_2HPO_4/NaH_2PO_4, 0.01 M Tris-HCl [pH 6.3], 10 mM β-ME) and buffer C with 0.2% Triton X-100, separately. Finally, the immune complexes were eluted by elution buffer (200 mM imidazole in 5% SDS, 0.15 M Tris-HCl [pH 6.7], 30% glycerol, 0.72 M β-ME) and analyzed by immunoblotting with indicated antibodies.

Immunofluorescence staining

Cells grown on glass coverslips were fixed with 4% paraformaldehyde for 20 min in dark, washed three times with PBS, and then permeabilized with 0.1% Triton X-100 and blocked with 1% bovine serum albumin for 1 h. Slices were incubated with the indicated primary antibodies overnight at 4 ℃, then followed by incubation with fluorescent-dye conjugated secondary antibodies. Nuclei were counterstained with DAPI (D1306, Invitrogen). The images were acquired by a Carl Zeiss laser-scanning confocal microscope with a 63X objective. Antibodies are listed as follows: Donkey anti-Rabbit IgG (H+L) Secondary Antibody, Alexa Fluor 555 (A31572, Thermo Fisher), Donkey anti-Mouse IgG (H+L) Secondary Antibody, Alexa Fluor 488 (A21202, Thermo Fisher).

Xenograft mouse model

Mice were purchased from the Laboratory Animal Center of Sun Yat-Sen University, and all animal experiments were performed according to the Institutional Animal Care and Use Committees of Sun Yat-Sen University. To generate murine subcutaneous tumors, indicated NCI-H1299 cells (5×10^6 cells per mouse) were injected subcutaneously into the right posterior flanks of 4-week-old immunodeficient nude mice. Once the tumors reached a volume of approximately 100 mm^3, mice were randomly allocated into groups (n = 5 mice/group) and treated with erastin (15 mg/kg intraperitoneal injection, twice every other day) via intraperitoneal injection for 20 days. Mice were measured every four days and volumes were calculated using the formula length $\times$ width^2 $\times$ $\pi/6$. Mice were then sacrificed; the tumor tissues were formalin-fixed and paraffin-embedded for histological analysis. The erastin was dissolved in 5% DMSO + 95% corn oil (C8267, Sigma-Aldrich).

Statistical analysis

All statistical analyses were performed using GraphPad Prism 7.0 software (GraphPad Software, Inc.). Data are presented as mean ± S.D. from three independent experiments. Unpaired Student's t test was used for statistical analysis between two groups. Multiple group comparisons were completed using one-way analysis of variance (ANOVA) or two-way ANOVA as appropriate. A p value < 0.05 was considered statistically significant.

189 **SUPPLEMENTARY FIGURES**

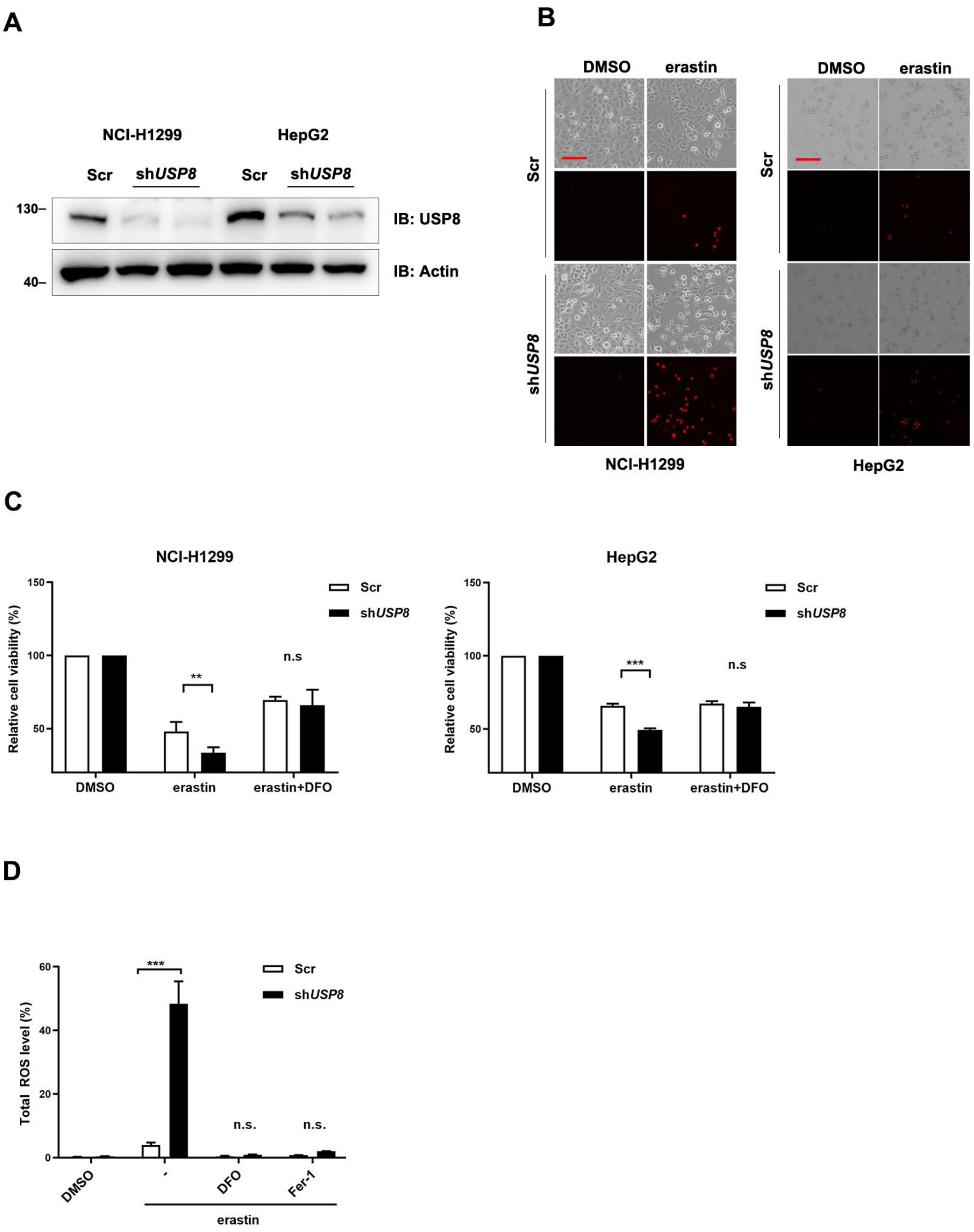

190

Figure S1 *USP8* knockdown increases erastin-induced ferroptosis. (A) Knockdown of *USP8* in NCI-H1299 and HepG2 cells was verified by immunoblotting. **(B)** NCI-H1299 cells and HepG2 cells were treated with 5 μM or 10 μM erastin for 24 h respectively, cell death was observed using PI staining by microscopy (scale bar = 100 μm). **(C)** NCI-H1299 cells and HepG2 cells were treated with 5 μM or 10 μM erastin alone or with DFO (20 μM) for 24 h, cell viability was assayed using a CCK8 kit. **(D)** Cells were treated with 1μM erastin alone or with a ferroptosis inhibitor (DFO, 10 μM; Fer-1, 2 μM) for 8h, the total ROS level was assessed by flow cytometry.

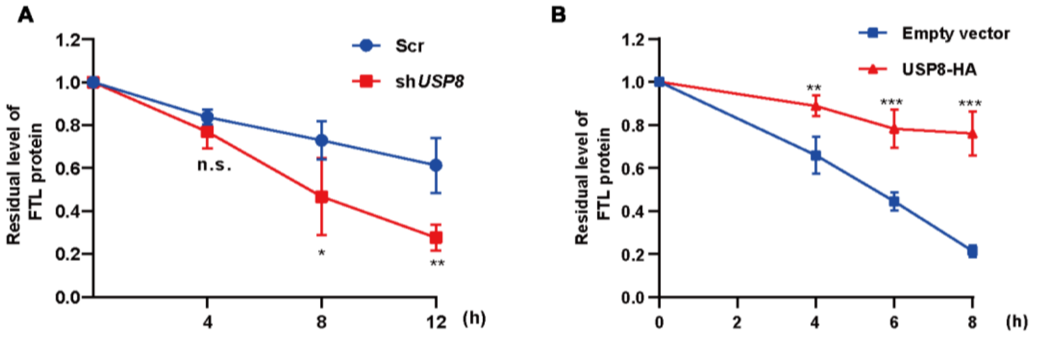

Figure S2 USP8 regulates ferritin degradation. (A) The relative band intensities of FTL in Figure 1N were quantitated using ImageJ and data were presented as mean ± S.D. from three independent experiments. **(B)**The relative band intensities of FTL in Figure 1O were quantitated using ImageJ and data were presented as mean ± S.D. from three independent experiments.

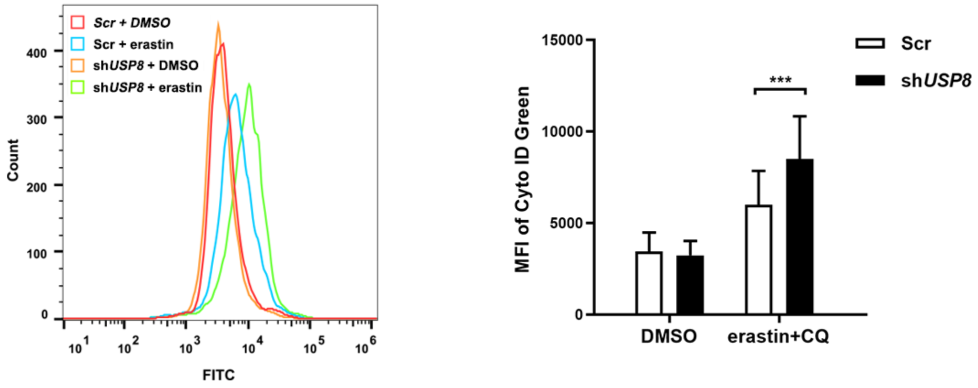

Figure S3 *USP8* knockdown increased autophagic flux. Indicated MEF cells were treated with 1 μM erastin for 8 h with or without CQ (50 μM, 6 h), then autophagy level was assessed by flow cytometry using a Cyto-ID autophagy detection kit.

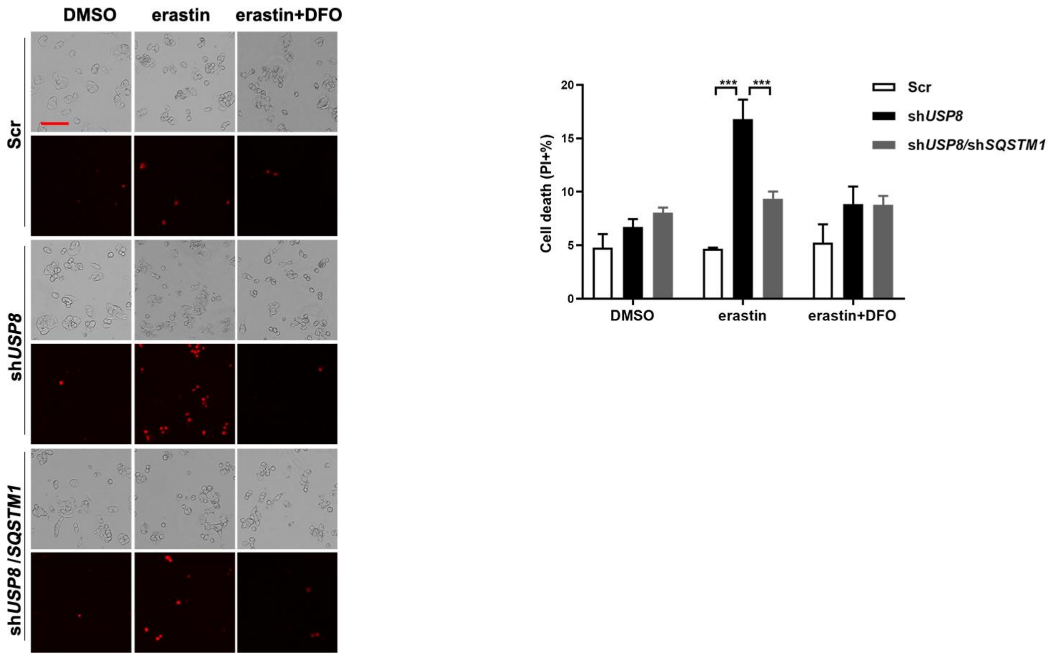

Figure S4 SQSTM1 is required for USP8-mediated ferroptosis. Indicated HepG2 cells were treated with erastin (10 μM) or with DFO, cell death was observed using PI staining by microscopy (black and white: phase contract; red: PI staining; scale bar = 100 μm) and quantified with flow cytometry.

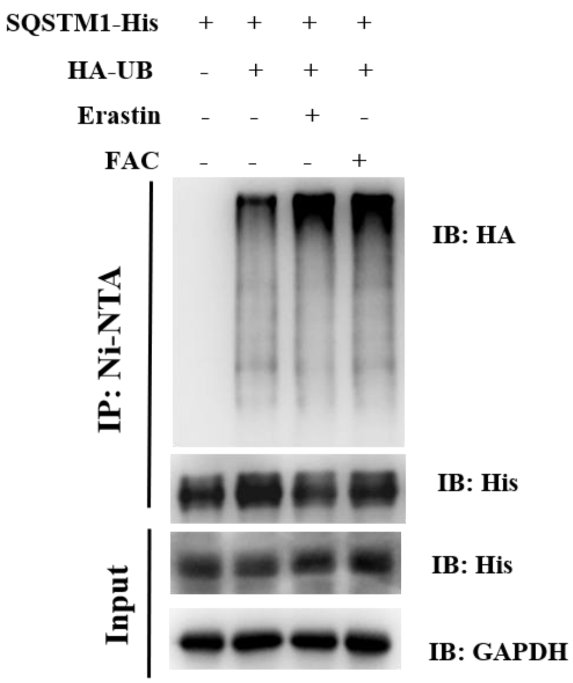

Figure S5 Ubiquitination of SQSTM1 was affected by iron level. HEK293T cells were co-transfected with SQSTM1-His, HA-UB for 24 h, followed by treatment with 10 μM erastin or 10 μM FAC for 12 h. The cell lysates were subjected to pull down using the Ni^{2+}-NTA beads under denaturation conditions.

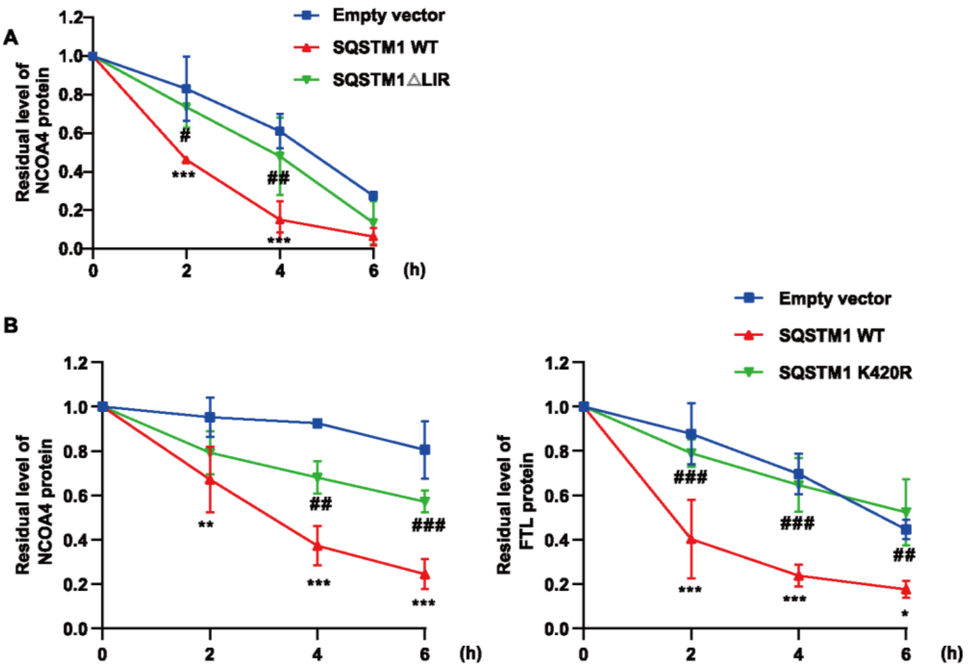

Figure S6 Protein levels of FTL and NCOA4 were both regulated by SQSTM1. (A) The relative band intensities of NCOA4 in Figure 2F were quantitated using ImageJ and data were presented as mean ± S.D. from three independent experiments. SQSTM1 WT vs. Empty vector: ***$p \leq 0.001$; SQSTM1-ΔLIR vs. SQSTM1 WT: $^{\#}p \leq 0.05$, $^{\#\#}p \leq 0.01$. **(B)** The relative band intensities of NCOA4 and FTL in Figure 2I were quantitated using ImageJ and data were presented as mean ± S.D. from three independent experiments. SQSTM1 WT vs. Empty vector: *$p \leq 0.05$, **$p \leq 0.01$, ***$p \leq 0.001$; SQSTM1 K420R vs. SQSTM1 WT: $^{\#\#}p \leq 0.01$, $^{\#\#\#}p \leq 0.001$.

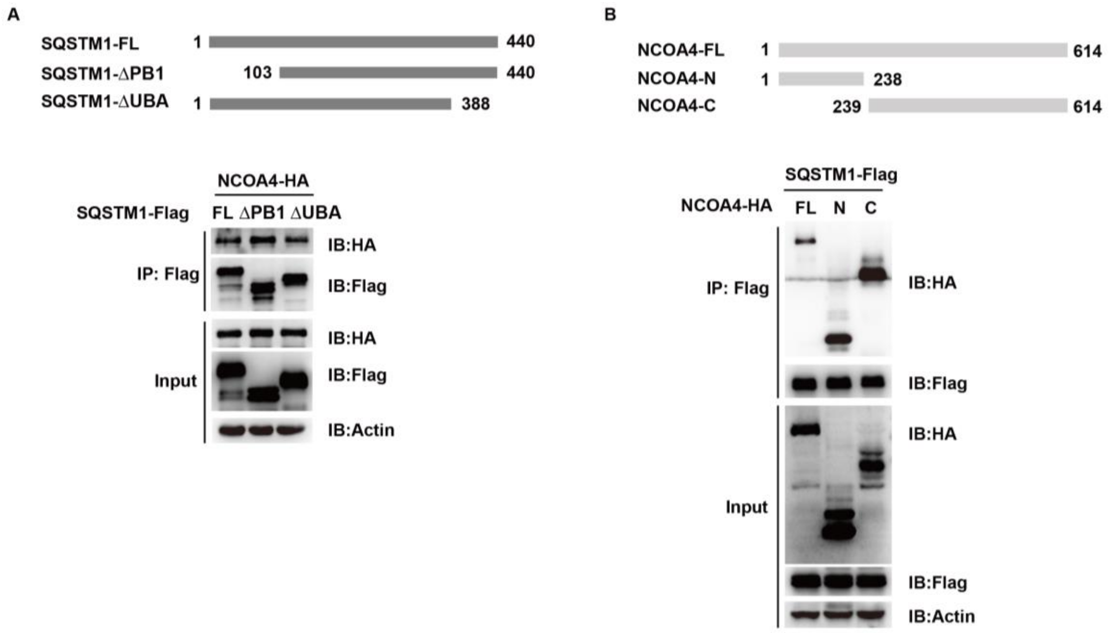

Fig. S7 Interactions between the truncation mutants of SQSTM1 and NCOA4. (A) Schematic representation of SQSTM1-Flag and its truncated mutants (upper panel). HEK293T cells were co-transfected with NCOA4-HA and SQSTM1-Flag or its truncated mutants, the cell lysates were immunoprecipitated with flag beads and immunoblotted with indicated antibodies (lower panel). (B) Schematic representation of NCOA4-HA and its truncated mutants (upper panel). HEK293T cells were co-transfected with SQSTM1-Flag and NCOA4-HA or its truncated mutants, the cell lysates were immunoprecipitated with flag beads and immunoblotted with indicated antibodies (lower panel).

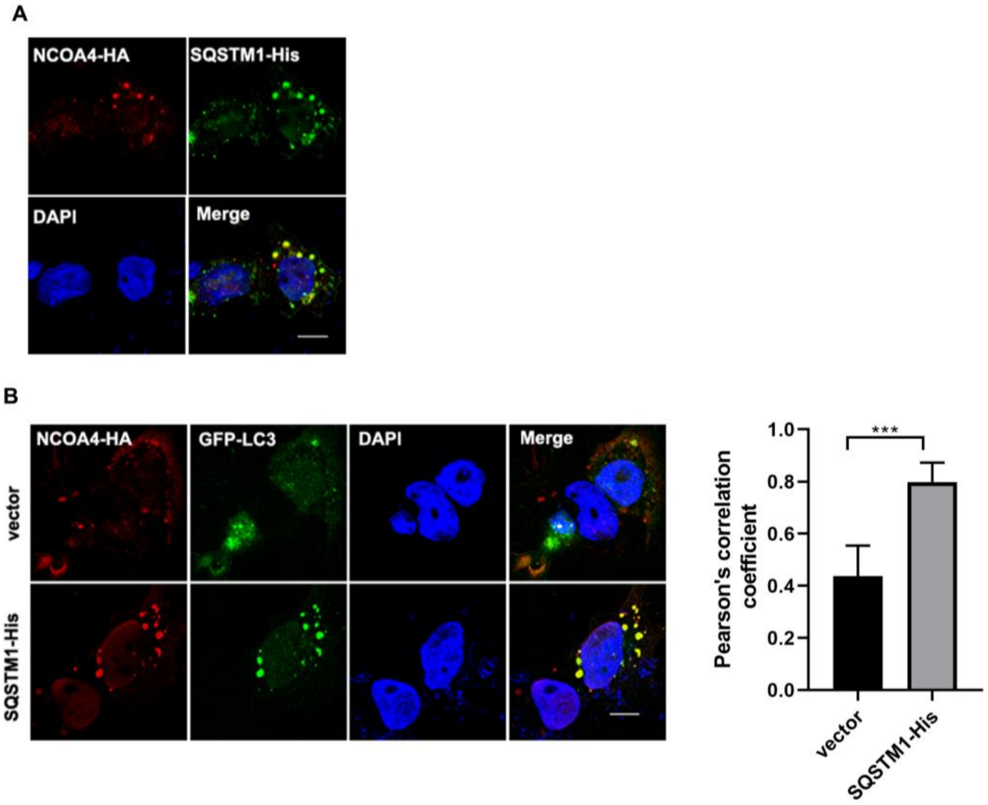

Figure S8 Immunofluorescence images showed NCOA4 colocalized with SQSTM1, and colocalized with LC3 much stronger in the presence of SQSTM1. (**A**) Hela cells grown on glass cover slips were transfected with NCOA4-HA and SQSTM1-His and treated with BAF (200 nM). Cells were then fixed with 4% PFA, processed for imaging, and visualized under the confocal microscope using the 63× magnification objective (scale bar = 10 μm). (**B**) Hela cells were transfected with NCOA4-HA and GFP-LC3 with or without SQSTM1-His, and treated with BAF (200 nM). Cells were then fixed, stained and visualized by confocal microscope (scale bar = 10 μm). Co-localization of NCOA4 and LC3 was analyzed by calculation of the Pearson's Correlation Coefficient. Bar graph showed mean ± S.D. from at least 25 cells in three independent samples.

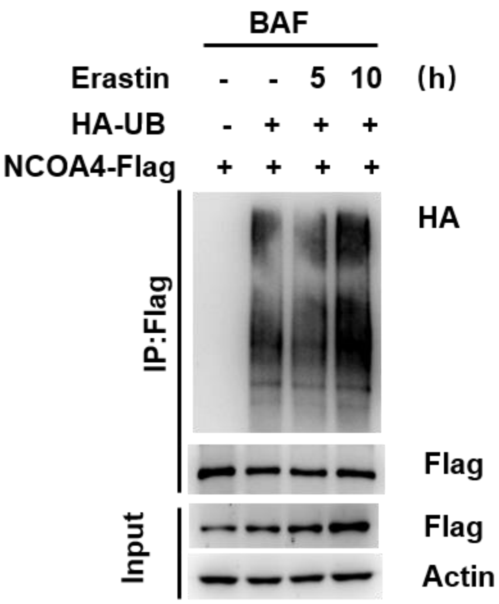

Figure S9 Erastin treatment enhanced the ubiquitination level of NCOA4. HEK293T cells were co-transfected with NCOA4-Flag and HA-UB for 24 h, followed by treatment with 5 or 10 μM erastin and BAF for 12 h. The cell lysates were subjected to pull down using the Flag beads, followed by immunoblotting.

SUPPLEMENTARY TABLE

Table S1. list of primers sequences used for qRT-PCR

**Gene**	**Sequence (5’-3’)**
human FTL	Forward: TACGAGCGTCTCCTGAAGATGC<br>Reverse: TCAGCTTTTTCTCCAGGGC
human GAPDH	Forward: AGTGGGTGTCGCTGTTGAAGT<br>Reverse: AACGTGTCAGTGGTGGACCTG
